# Increased Exosomal MicroRNA-21 and MicroRNA-146a Levels in the Cervicovaginal Lavage Specimens of Patients with Cervical Cancer

**DOI:** 10.3390/ijms15010758

**Published:** 2014-01-08

**Authors:** Jie Liu, Hong Sun, Xiaoli Wang, Qun Yu, Shuhong Li, Xiaoyan Yu, Wenwen Gong

**Affiliations:** Department of Obstetrics and Gynecology, Yantai Yuhuangding Hospital of Qingdao University, Yantai 264000, Shandong, China; E-Mails: jliuyt2009@163.com (J.L.); hsunyt2013@163.com (H.S.); qyuyt2006@163.com (Q.Y.); shuhongli2005@163.com (S.L.); yuxyyt2013@163.com (X.Y.); wenweng2000@163.com (W.G.)

**Keywords:** cervical cancer, exosomes, microRNA-21, microRNA-146a

## Abstract

Well-run screening programs for cervical cancer in the population at risk have been shown to result in a sharp decrease in the incidence and mortality of cervical cancer in a number of large populations. Expression patterns of a recently identified biomarker family, microRNA, appear to be characteristic of tumor type and developmental origin. Several tumors have been reported to actively release exosomes carrying microRNAs. The present study has determined the association of microRNAs with cervical cancer-derived exosomes. The cervical cancer-derived exosomes were enriched in the cervicovaginal lavages specimens and the abundance of exosomes and exosomal microRNAs was detected by electron microscopy, western blot analysis, RT-qPCR and microRNA target reporter vector. The microRNA-21 and microRNA-146a, which were up-regulated in cervical cancer patients, were associated with the high levels of cervical cancer-derived exosomes. In conclusion, we demonstrated the abundance of exosomes in the cervicovaginal lavage specimens of women with cervical cancer. Furthermore, our results indicated that abnormally high levels of microRNA-21 and microRNA-146a existed in the cervical cancer-derived exosomes and the two microRNAs were functional in 293T cells.

## 1. Introduction

Cervical cancer (CC) is one of main causes of cancer-related death in women [1] and the third most common cancer among women worldwide [2]. Persistent cervical infection with high-risk HPV genotypes contributes to the development of cervical cancer [3], the cervical intraepithelial neoplasia (CIN) [4], and the pre-cancer of the cervix. Epidemiologic data have shown that nearly 100% of cervical cancer cases test positive for HPV [5]. The most carcinogenic HPV type 16 (HPV16) and the second most carcinogenic HPV18 account for more than 60% of all cervical cancers [6,7]. The establishment of the causal link between HPV and cervical cancer, along with an understanding of the HPV infection history, have refreshed the model for cervical carcinogenesis: persistent HPV infection progresses to pre-cancer and eventually causes cancer (*in situ* and invasive) [8]. Despite the robust carcinogenic potential, HPV infection alone is not sufficient for the development of cervical cancer, because only a minor fraction of patients infected with HPV develop cervical cancer [8]. Indeed, several cofactors, including dysregulation of microRNAs, have been implicated in the genesis of HPV-associated cervical cancer [9,10].

MicroRNAs (miRNAs) are non-coding RNA molecules of approximately 22 nucleotides that regulate gene expression in organisms ranging from nematodes to humans [11], and in a broad array of cell processes in mammals [12–14]. Recent years, numerous oncogenic microRNAs have been reported to be associated with cervical cancer tumorigenesis [15,16]. MicroRNA-10a, microRNA-21, microRNA-19, and microRNA-146a promote cell growth, migration and invasion in human cervical cancer cells [9,10,17]. Conversely, tumor suppressive microRNAs have been down-regulated in human cervical cancer, such as microRNA-372, microRNA-214, and microRNA-218 [18–20]. In addition, there are numerous other microRNA dysregulation in the human cervical cancer. MicroRNA-34a suppresses invasion through down-regulation of Notch1 and Jagged1 in cervical carcinoma and choriocarcinoma cells [21]. The microRNA-302–367 cluster suppresses the proliferation of cervical carcinoma cells through the novel target AKT1 [22]. miRNA expression profiles have been shown to be promising biomarkers for the diagnosis, classification or outcome prediction of a wide array of human cancers (reviewed in refs. [23,24]). The clinical value of the above-mentioned miRNAs as markers for cervical cancer needs to be further investigated.

The majority of microRNAs are present within the cell, and only a smaller number of them have been detected outside cells, including various body fluids [25–27]. Surprisingly, extracellular microRNAs are remarkably stable despite high extracellular RNase activity [27], indicating that these microRNAs are likely packaged to avoid RNase digestion. Recent studies have indeed confirmed that extracellular microRNAs are shielded against degradation by packaging in one type of extracellular vesicles, exosomes [25,28]. Exosomes are first discovered in the maturing mammalian reticulocyte [29], and are later found to be present in many and perhaps all biological fluids, including urine, blood, ascites, cervical secretions, etc., with the diameter between 30 and 110 nm and the density ranging from 1.13 to 1.19 g/mL [30,31]. The exosome has been implicated in intercellular communication via cargos of proteins, mRNAs, and miRNAs [32]. More recently, the exosomal microRNAs are used as diagnostic biomarkers for lung cancer, ovarian cancer, and cardiovascular diseases [33–36].

The present study is to determine whether the cervical cancer-derived exosomes contain abnormally high levels of microRNA-21 and microRNA-146a, which are up-regulated in cervical cancers, and to evaluate whether the exosomal microRNA-21 and microRNA-146a can potentially serve as useful biomarkers for cervical cancer diagnosis.

## 2. Results

### 2.1. Abundance of Exosomes in the Cervicovaginal Lavage Specimens of Women with Cervical Cancer

**Figure 1.**
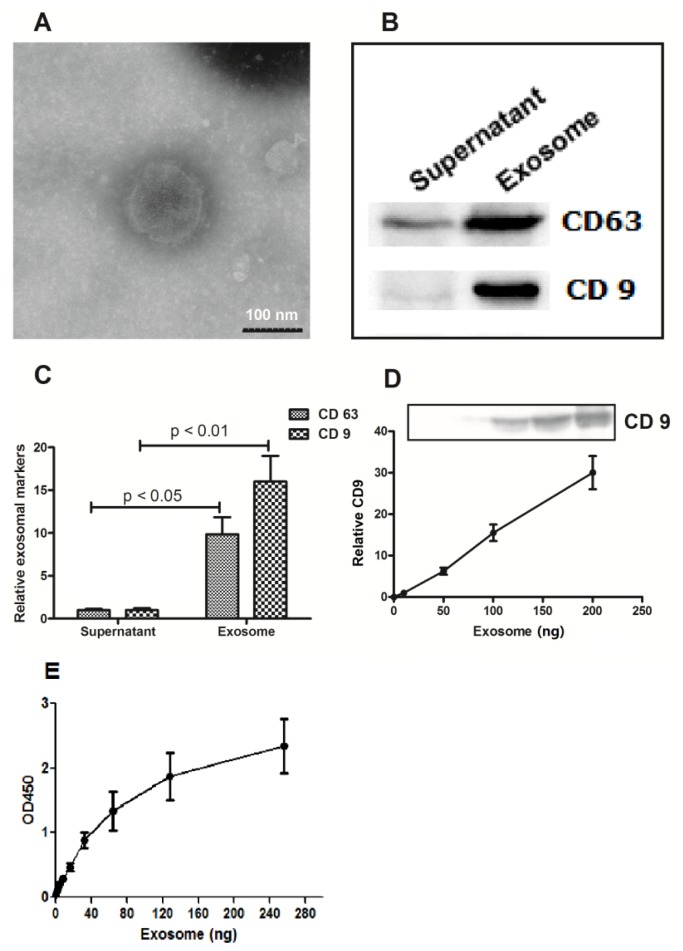
Confirmation of the exosomal vesicles in the cervicovaginal lavage specimens. (**A**) Electron microscopy of the vesicles from cervicovaginal lavage specimens revealed the characteristic spherical shape and the size (40–100 nm; sized from 10 vesicles) of exosomes; (**B**,**C**) Western blot showed strong staining of the ultracentrifugation pellets with the antibodies against the exosomal membrane markers, CD63 and CD9; (**D**,**E**) Dose-dependent correlation between the CD9 expression level and the presence of exosomes isolated from the cervicovaginal lavage specimens was tested by western blot (**D**) and by ELISA (**E**). The exosome samples were centrifuged at 13,500× *g* for 30 min at 4 °C, and ultracentrifuged at 100,000× *g* for 2 h at 4 °C before analysis.

Cervicovaginal lavages specimens were collected from 45 patients with cervical cancers, 25 HPV-positive subjects and 32 normal HPV-negative subjects. Exosomes were isolated from the specimens by ultracentrifugation. The exosomes were examined and confirmed by electron microscopy. Under electron microscopy, the pellets were spherical in shape, with an average diameters varying between 30 and 110 nm (Figure 1A), consistent with the previously reported characteristics of exosomes [37]. The exosomal vesicles were further confirmed by western blot analysis using antibodies against two common exosomal markers, the tetraspanin molecules CD63 and CD9 [38,39] (Figure 1B,C). The expression level of CD9 was quantitatively measured in the exosomes (Figure 1D,E).

### 2.2. Significant High Levels of MicroRNA-21 and MicroRNA-146a in Cervical Cancer-Derived Exosomes

**Figure 2.**
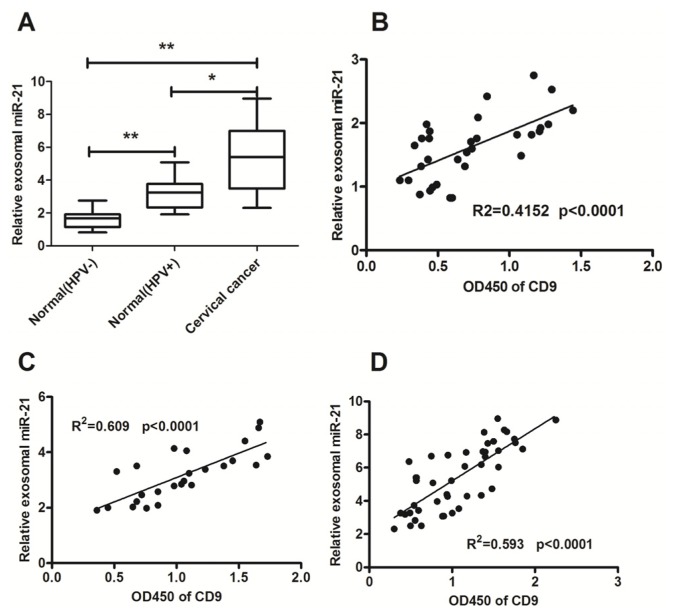
Enriched exosomal microRNA-21 in cervicovaginal lavage specimens of cervical cancer patients. (**A**) The relative amounts of exosomal miR-21 were examined by RT-qPCR in cervicovaginal lavage specimens of the normal subjects with HPV-negative (*n* = 32), subjects with HPV-positive (*n* = 25), or cervical cancer patients (*n* = 45). The lowest expression in the normal HPV-negative group was designated as “1”. All other samples were expressed as a relative value according to the ratio of their Cp value to the Cp value of the designated sample; (**B**–**D**) The correlation between the miR-21 expression and the levels of exosomes was evaluated by RT-qPCR and ELISA; (**B**) HPV-negative normal group; (**C**) HPV-positive group; (**D**) Cervical cancer group. All experiments were performed in triplicate. Statistical significance was considered with a *p* < 0.05 (*) or *p* < 0.01 (**).

**Figure 3.**
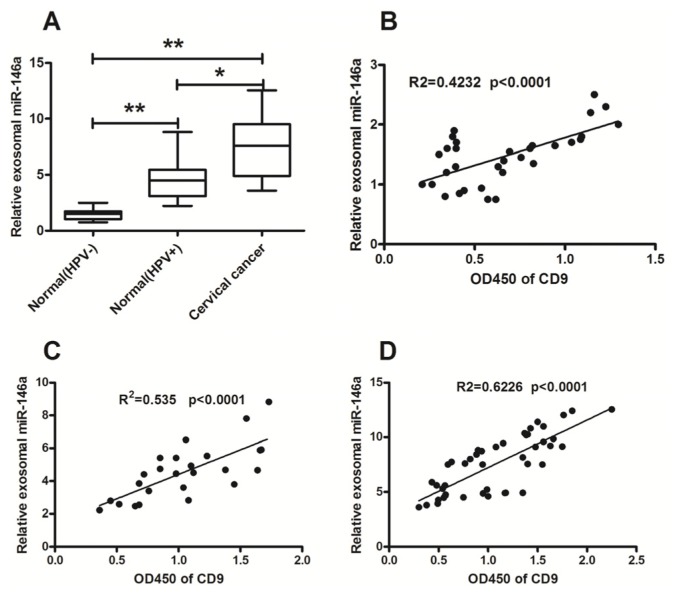
Enriched exosomal microRNA-146a in cervicovaginal lavage specimens of cervical cancer patients. (**A**) The relative amounts of miR-146a were examined by RT-qPCR in cervicovaginal lavage specimens of the HPV-negative normal subjects (*n* = 32), the HPV-positive subjects (*n* = 25), or the cervical cancer patients (*n* = 45). The lowest expression in the normal (HPV-) group was designated as “1”. All other samples were expressed as a relative value according to the ratio of their Cp value to the Cp value of the designated sample; (**B**–**D**) The correlation between the miR-146a expression and the level of exosomes was evaluated by RT-qPCR and ELISA; (**B**) HPV–negative normal group; (**C**) HPV-positive group; (**D**) Cervical cancer patient group. All experiments were performed in triplicate. Statistical significance was considered with a *p* < 0.05 (*) and a *p* < 0.01 (**).

Based on previous reports, microRNA-21 and microRNA-146a were chosen to be investigated in this study (Table 1) [10,40–44]. These two microRNAs have been demonstrated to be involved in oncogenesis in cervical cancer (Table 1). The exosomal microRNA-21 and microRNA-146a in cervicovaginal lavage were evaluated in 25 HPV-positive subjects, 32 HPV-negative normal subjects, and 45 patients with cervical cancer in this study. The expression of miRNA-21 in cervicovaginal lavage specimens of the cervical cancer subjects was significantly higher than that of the HPV-positive subjects and HPV-negative normal subjects (Figure 2A) (both *p* < 0.01). The HPV infection seemed to contribute to the up-regulation of microRNA-21 in cervical tissue [43]. Differential exosomal microRNA-21 expression was also found between the HPV-positive and HPV-negative normal groups (Figure 2A) (*p* < 0.05). The exosomal miRNA-146a level in cervicovaginal lavage specimens of the cervical cancer patients was also significantly higher than that of the HPV-positive subjects and HPV-negative normal subjects (Figure 3A) (both *p* < 0.01). However, there was no significance in the miRNA-21 or miRNA-146a level in the supernatant of cervicovaginal lavage among the three groups. In addition, the relative miRNA-21 or miRNA-146a level in exosomes was significantly higher than in the supernatant (Supplementary Figure S1).

The correlation between the levels of the exosomal microRNA-21 or microRNA-146a in cervicovaginal lavage specimens and the levels of the exosome marker CD9 was calculated using the SPSS for Windows software version 11.0. The exosomal CD9 in all cervicovaginal lavage specimens was determined by an ELISA assay. The microRNA-21 and microRNA-146a levels in the cervicovaginal lavage specimens of cervical cancer patients or control groups were all correlated with the exosomal CD9 (Figure 2B–D; Figure 3B–D). Therefore, the significant high level of microRNA-21 and microRNA-146a existed mainly in the exosomes secreted in the cervicovaginal lavage specimens.

### 2.3. MicroRNA Can Be Released by Stimulation with a Calcium Ionophore

**Figure 4.**
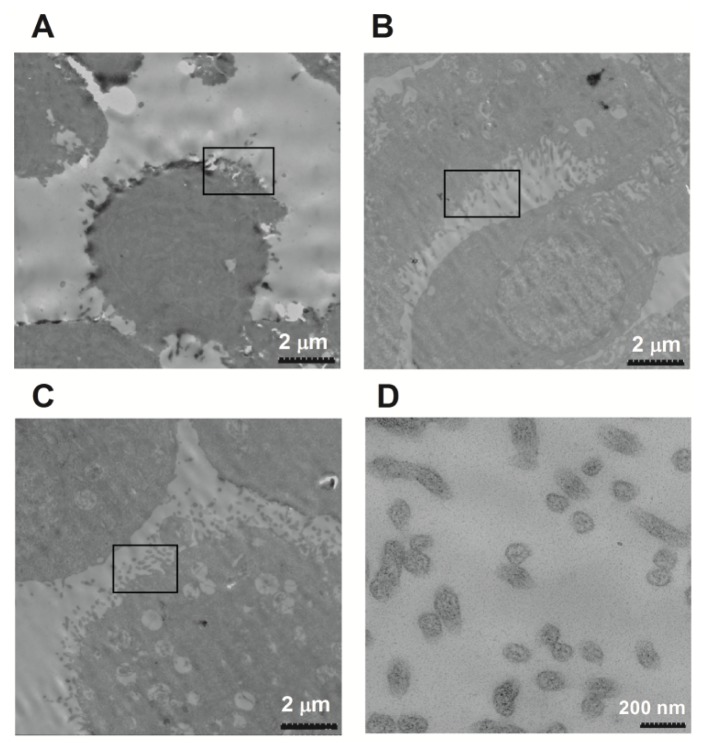
Electron microscopy images of increased exosome release from Hela cells after the stimulation with a calcium ionophore. (**A**) Hela cells without A23187 stimulation; (**B**) Hela cells stimulated with 0.5 μmol/L of A23187; (**C**,**D**) Hela cells stimulated with 1 μmol/L of A23187.

The results in Figures 2 and 3 showed up-regulation of microRNA-21 and microRNA-146a in the cervicovaginal lavage specimens of cervical cancer patients. It is also known that exosomes and exosomal microRNAs are released after the stimulation with Ca^2+^ [28,45,46]. Thus, we examined whether exosomes were increasingly released from the cervical adenocarcinoma Hela cells after being stimulated for 1 h with A23187, a calcium ionophore. Figure 4 indicated that under electron microscopy, more exosomes were released from the Hela cells after the treatment with A23187 at concentrations of 0.5 or 1 μmol/L, compared to non-treated cells.

**Figure 5.**
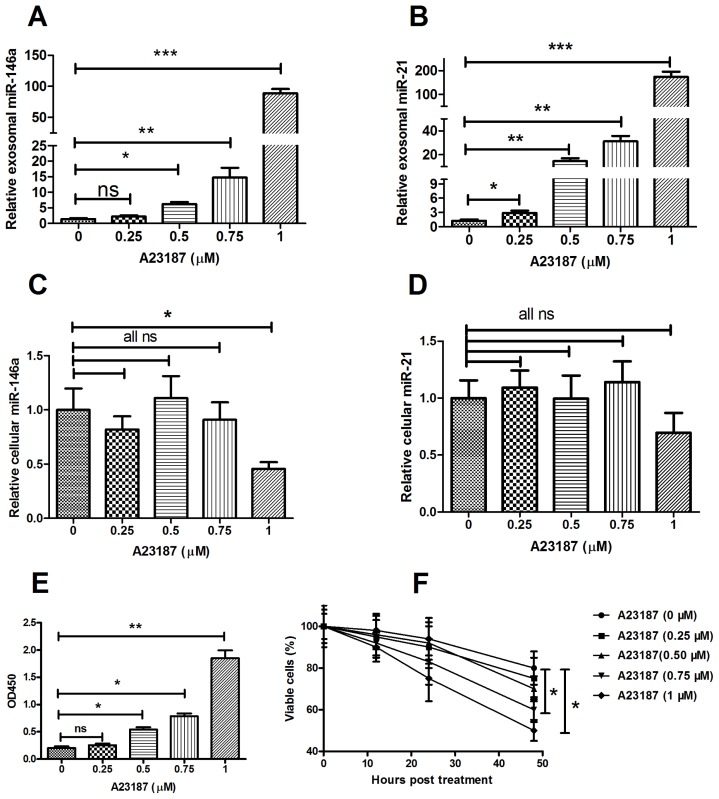
Calcium ionophore stimulated exosome secretion in Hela cells. (**A**,**B**) Relative expression levels of microRNA-146a (**A**) and microRNA-21 (**B**) in the exosomes released from Hela cells after the stimulation with various concentrations of A23187 for one hour; (**C**,**D**) Cellular microRNA-146a (**C**) and microRNA-21 (**D**) in Hela cells after the A23187 treatment for one hour; (**E**) Exosome were quantified by ELISA For CD9; (**F**)Viability of the Hela cells after the A23187 treatment. All the exosome samples released from cells were quantified by the ELISA for CD9 before miRNA assay. In addition, all experiments were performed in triplicate. Statistical significance was considered with *p* < 0.05 (*), *p* < 0.01 (**) and *p* < 0.001 (***). ns, no significance.

After the Hela cervical cancer cells were treated with A23187, the release of microRNA-21 and microRNA-146a were determined as well. Exosomes were collected from the equal volumes of culture medium of the Hela cells with or without the A23187 stimulation, and the levels of microRNA-21 and microRNA-146a were measured by RT-qPCR. In Figure 5, the release of microRNA-146a (Figure 5A) and microRNA-21 (Figure 5B) from Hela cells into the culture medium were induced by the A23187 treatment in dose-dependent manner. The A23187 treatment did not significantly alter the expression levels of those two microRNAs inside the Hela cells, except that microRNA-146a showed reduction in Hela cells treated with 1 μM of A23187. This suggested that A23187 did not regulate the microRNAs expression (Figure 5C,D) and that the high concentration of A23187 decreased cell viability (Figure 5E).

### 2.4. Exosomal MicroRNA-21 Was Functional and Taken Up by Recipient Cells

**Figure 6.**
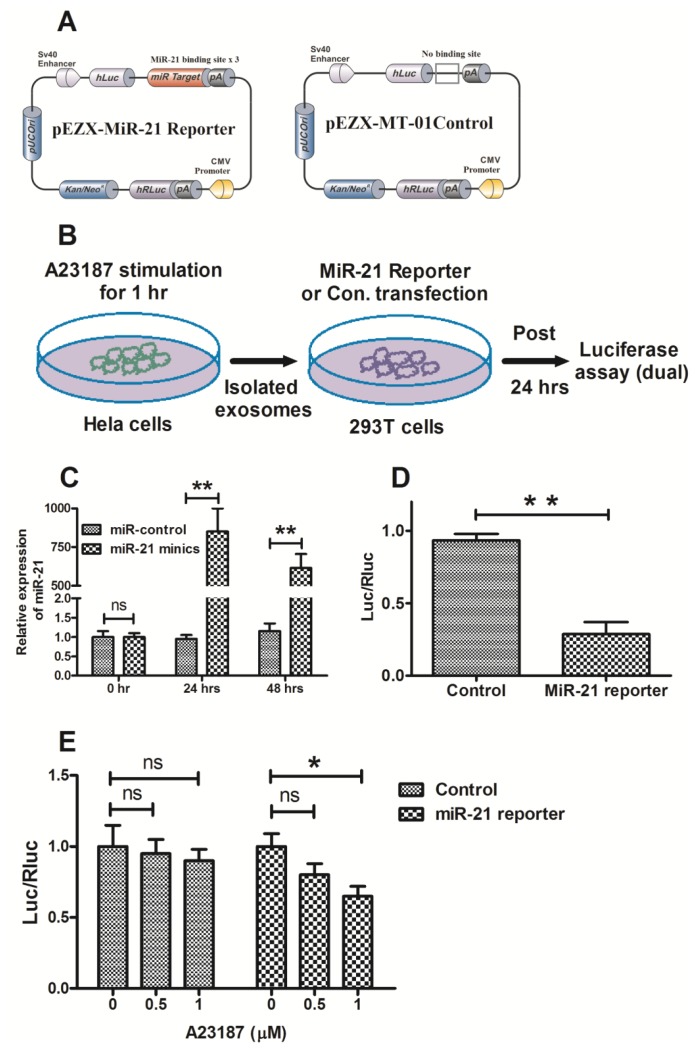
Exosomal microRNA-21 was functional in recipient cells. (**A**) Schematic diagram of pEZX-MT01 plasmid containing luciferase reporters with or without the target sequence of microRNA-21; (**B**) Schematic for detecting the function of microRNA-21 in the released exosomes; (**C**) The expression of microRNA-21 was significantly up-regulated in 293T cells after transfection with the microRNA-21 mimics; (**D**) Relative luciferase activity of miR-21 sensor reporter or sensor control in 293T cells transfected with the microRNA-21 mimics; (**E**) Exosomal microRNA-21 from Hela cells significantly reduced the luciferase activity of the sensor reporter in 293T cells. All the exosome samples released from cells were quantified by the ELISA for CD9 before miRNA assay. In addition, all experiments were performed in triplicate. Statistical significance was considered with *p* < 0.05 (*) and *p* < 0.01 (**). ns, no significance.

To further validate the exosomal microRNA release induced by the calcium ionophore, A23187, we examined the function of exosomal microRNA-21 from Hela cells using a luciferase reporter plasmid containing a target sequence of microRNA-21, as indicated in the Materials and Methods section (Figure 6A,B). The effectiveness of the reporter vector was first evaluated in the 293T cells transfected with microRNA mimics. After transfection with the microRNA-21 mimics, the RNA levels of microRNA-21 were increased about 800-fold in the 293T cells (Figure 6C), and the ratio of firefly to Renilla luciferase activity was significantly decreased (Figure 6D). In addition, the relative luciferase activity was reduced in the 293T cells inoculated with the A23187-induced exosomes (Figure 6E).

## 3. Discussion

MicroRNA expression is dysregulated in a variety of diseases, and has also been shown to be a promising biomarker for diagnosing human cancers. While the stability of microRNAs is crucial for their biological effect, very little is known regarding the post-biogenesis or post-maturation regulation of microRNAs. In contrast to their precursors, pri-microRNAs, mature microRNAs appear to have the 5′- and 3′-unprotected ends that may render them accessible to exoribonucleases. In general, microRNAs do not necessarily exert their main functions in the cells where their synthesis takes place. It has been speculated that considerable amounts of microRNAs should be packed into vesicles [47]. Recent studies have actually confirmed that extracellular microRNAs are shielded against degradation by packaging in exosomes [25,28]. Rabinowits G *et al*. found significant difference in the total circulating exosomes and the miRNA levels between lung adenocarcinoma patients and controls, demonstrating the use of circulating exosomal miRNAs as a screening test for lung adenocarcinoma [34]. The microRNA profiling of circulating exosomes of ovarian tumors also indicated the potential use of microRNAs as diagnostic markers for asymptomatic cancer patients [35]. In addition, the elevated levels of circulating exosomal miR-133a have been observed in patients with cardiovascular diseases mainly in the injured myocardium [36]. Furthermore, it was found that the majority of the detectable microRNAs in serum and saliva were concentrated in exosomes [33]. Therefore, the utilization of exosomal microRNAs as diagnostic biomarkers of cancers and other diseases needs to be further determined.

In this study, we examined the morphology of exosomes isolated from cervicovaginal lavage specimens of cervical cancer patients by electron microscopy, and measured the expression of exosome-specific markers (CD9 and CD63) by Western blot analysis. The electron microscope images indicated that the exosomes were spherical or cup-shaped, with a diameter of 30–100 nm. The exosome-specific markers, CD9 and CD63, existed primarily in the vesicles collected by ultracentrifugation, rather than the supernatant of the cervicovaginal lavage specimens. The RNA levels of microRNA-21 and microRNA-146a were both elevated in the enriched exosomes from the cervical cancer patients. The exosomal microRNAs were further investigated by using a calcium ionophore (A23187) as well as a microRNA-21 sensor reporter vector. MicroRNA-21 and microRNA-146a released from a cervical adenocarcinoma cell line, Hela, were significantly up-regulated after the stimulation with A23187. The exosomes released by Hela cells reduced the luciferase activity of the microRNA-21 sensor reporter in 293T cells. Taking together, this study reports for the first time the elevated levels of exosomal microRNAs in cervicovaginal lavage specimens of cervical cancer patients, implying a potential application in non-invasive cervical cancer screening. Well-run screening programs for cervical cancer in the population at risk have been shown to result in a sharp decrease in the incidence and mortality of cervical cancer in a number of large populations [48–51]. The Papanicolaou test (*i.e.*, Pap smear, Pap test, or smear test) is used to screen potentially pre-cancerous and cancerous processes in the endocervical canal, with a sensitivity of about 68% and a specificity of about 75% [52]. The accuracy of the Pap test is certainly influenced by the adequacy of the specimen obtained and personnel training [53]. Newer techniques such as liquid-based cytology showed a modestly higher sensitivity for detecting any degree of CIN and a modestly lower specificity [54]. The HPV test is an alternative option for women who are at higher risk for cervical cancer and who need to be screened more often, particularly for women older than age 30 [55]. Obviously, more promising diagnostic methods with higher sensitivity and specificity are needed for the cervical cancer screening.

Expression profiling of microRNAs has been shown to be highly specific for the types and origin of cancers, as well as the progression of diseases. However, it is difficult to detect the microRNA profile of abnormal tissues without invasive procedure, and the microRNA levels from the abnormal tissues were usually not enough to cause changes in the level of circulating microRNAs. Therefore, it may be a feasible approach to detect the abnormal microRNAs in secretions from the primary tumor site, particularly for epithelium-derived cancers, such as cervical cancers, nasopharyngeal carcinomas, lung cancers, gastric cancers, carcinoma of colon, and so on. In this study, we determined the levels of microRNA-21 and microRNA-146a in the supernatant of cervicovaginal lavage specimens after ultracentrifugation, and no statistical significance of the two microRNAs was observed between cervical cancer and normal groups (Supplementary Figure S1). Surprisingly, the microRNA levels were significantly higher in the vesicles from the lavage specimens of cervical cancer patients and subjects with HPV-positive. The high levels of microRNAs were directly correlated with the expression level of CD9, an exosome marker. Thus, we deduced that the cervical cancer-associated microRNA-21 and microRNA-146a were secreted from the cancer tissue to the cervicovaginal cavity with the exosomes. Abnormality of the microRNAs in the enriched cervicovaginal exosomes might be an indication of cervical cancer.

## 4. Materials and Methods

### 4.1. Research Subjects

Forty-five patients with cervical cancer of stage IB–IIB (ALL squamous cell carcinoma; 25 cases were HPV16-positive and 20 cases were HPV18-positive), 25 normal subjects with HPV-positive (HPV18) and 32 normal subjects with HPV-negative were enrolled in this study. There was no cervical (pre) cancerous disease in all normal subjects (with or without HPV infection), screened by liquid-based cytology. Cervicovaginal lavage fluid from the 45 patients was collected prior to any treatment. The normal control cervicovaginal lavage fluid was collected from volunteers with or without HPV infection. Written informed consent was obtained from all of the subjects for using their cervicovaginal lavage fluid for research purposes. The use of clinical material was approved by the institutional review board according to the guidelines of the Dutch Federation of Medical Research Associations.

### 4.2. Reagents and Cell Lines

Hela cell line was purchased from ATCC and maintained in Dulbecco’s modified Eagle’s medium (DMEM; Invitrogen, Carlsbad, CA, USA) supplemented with 10% fetal bovine serum (FBS; Invitrogen, Carlsbad, CA, USA) at 37 °C with 5% CO2. Calcium Ionophore A23187 (Sigma-Aldrich, St. Louis, MO, USA) was dissolved in DMSO with a concentration of 0.5 mM, and stored at −20 °C before use. CD9 and CD63 rabbit polyclonal antibodies were purchased from Santa Cruz Biotechnology (Santa Cruz, CA, USA); the goat anti-rabbit secondary antibody was purchased from Sino Biological (Beijing, China). The Reporter pEZX-MT-01 plasmid with microRNA-21 targets and the pEZX-MT-01 control plasmid were purchased from GeneCopoeia, Inc. (Rockville, MD, USA).

### 4.3. Exosome Isolation from Cervicovaginal Lavage Specimens or Culture Supernatant

PBS (10 mL) was utilized for the vagina and cervix lavage [56]. The lavage fluid converging at the posterior fornix was collected for exosome isolation by differential centrifugation. Exosomes from the supernatants of Hela cell culture were prepared as described previously [57]. Briefly, after the cells were washed three times with PBS, cell culture medium was replaced with serum-free medium, supplemented with 0, 0.5 or 1 μM A23187 at 24 h before exosome isolation. Cell culture supernatants were collected for exosomes isolation by differential centrifugation. First, the lavage fluid or the culture supernatants were centrifuged at 3000× *g* for 10 min at 4 °C to remove the cells and debris, and then was centrifuged at 13,500× *g* for 30 min at 4 °C, followed by ultracentrifugation at 100,000× *g* for 2 h at 4 °C. The pellet was stored at −80 °C for subsequent applications.

### 4.4. Scanning Electron Microscope

The ultracentrifuged pellets from cervicovaginal lavage specimens were resuspended in TBS and applied to a grid covered with collodion, then negatively stained with 2% phosphotungstic acid (Nisshin EM, Tokyo, Japan). The microphotographs were obtained using an HD-2000 scanning transmission electron microscope (Hitachi, Tokyo, Japan).

### 4.5. Immunological Assay for CD9 and CD63

The vesicles were resuspended in cell lysis reagent (Promega, Madison, WI, USA), and supplemented with protease inhibitor cocktail (Roche Biochemicals, Basel, Switzerland). After protein concentration determination using the BCA Protein Assay Reagent Kit (Pierce, Rockford, IL, USA), equal amounts of protein and sample buffer were separated in a 12% gradient SDS-PAGE gel; the gel was stained with Coomassie blue, or transferred to polyvinylidene fluoride membrane. The blotted membrane was blocked with tris-buffered saline containing 5% milk and incubated with the CD63 or CD9 rabbit polyclonal antibody (1:500; Santa Cruz Biotechnology, Santa Cruz, CA, USA), followed by incubation with horseradish peroxidase-coupled secondary antibody (1:1000; Cell Signaling Technology, Inc. Danvers, MA, USA). The proteins were detected using enhanced chemiluminescence (Thermo Scientific, Rockford, IL, USA). All immunoblots are representative of at least three independent experiments. ELISA was used to measure the exosome membrane-anchored CD9. The limit of detection was 25 pg/mL. 96 well-plates (Nunc, Milan, Italy) were coated with anti-CD63 polyclonal antibody (1:2000; Santa Cruz Biotechnology, Santa Cruz, CA, USA) and blocked by PBS containing 0.5% BSA. Purified exosomes from the cell culture supernatants or from the cervicovaginal lavage specimens were detected by anti-CD9 Mab (1:2000; Santa Cruz Biotechnology, Santa Cruz, CA, USA) according to manufacture instruction, and optical densities were read at 450 nm.

### 4.6. miRNA Isolation, RNAse Digestion, Reverse Transcription, qPCR

Exosomal miRNAs were isolated using mirVana miRNA Isolation Kit (Ambion, Austin, TX, USA) post the RNAse A (QIAGEN GmbH, Hilden, Germany) digestion of miRNAs outside exosomes. Reverse transcription (RT) reactions and quantitative polymerase chain reactions (qPCR) were performed using the MicroRNA TaqMan Reverse Transcription Kit and the TaqMan MicroRNA Assays (Applied Biosystems, Foster City, CA, USA) for mir-21 or mir-146a. Three microliters of cDNA were used as a template in a 15-μL PCR reaction. The template cDNA was amplified using specific primers for microRNA-21 or mocroRNA-146a. PCR reactions for each sample were run in triplicate.

### 4.7. Luciferase Activity Assay

Reporter plasmid with microRNA-21 sensitive sites (pEZX-MT01) was purchased from GeneCopoeia, Inc. (Rockville, MD, USA). Briefly, 1 × 10^5^ 293T cells were transfected with 50 nM of miRNA control or miR-21 mimics, and 0.5 μg of pEZX-MT01 reporter plasmid with Lipofectamine 2000 (Invitrogen, San Diego, CA, USA). The medium was replaced with DMEM containing 10% FBS at 6 h post-transfection. Cells were harvested at 24 h post-transfection for analysis. Exosomes induced by A23187 (0, 0.5 or 1 μM) from Hela cells (60 mm cell culture dish, 21 cm^2^) were isolated as mentioned above, and inoculated 293T cells (60 mm cell culture dish, 21 cm^2^) with a 3-mL volume. At 2 h post inoculation, cells were transfected with 0.5 μg pEZX-MT01 reporter or control plasmid. Cells were harvested at 24 h after transfection. Luciferase activity was determined by using a Promega luciferase assay system (IBM SPSS Inc. Armonk, NY, USA). Experiments were conducted in triplicate, and means were determined.

### 4.8. MTT Assay

Cell viability was determined by methyl thiazolyl tetrazoliym assay (MTT). Hela cells were plated in 96-well plates and grown up to 85%–90% confluency. Hela cells were then treated with 0, 0.25, 0.5, 0.75 or 1 μM A23187 for 0, 12, 24 or 48 h. After that, the medium was replaced with 50 μL MTT solution, and the cells were incubated at 37 °C for 2 h. The MTT solution was then discarded and 150 μL DMSO was added to dissolve the precipitate completely at room temperature. The optical density was measured at 570 nm using a spectrophotometer (Crystaleye, Olympus, Tokyo, Japan).

### 4.9. Statistical Analysis

The results are expressed as mean ± standard deviation. Student *t* test were performed to compare the differences between two groups. Correlation of the expression levels of microRNA-21, or microRNA-146a with the level of exosomal CD9 was calculated with the pearson correlation analysis of SPSS 17.0 software (IBM SPSS Inc. Armonk, NY, USA). Statistical significance was considered with *p* < 0.05 and *p* < 0.01.

## 5. Conclusions

In summary, we found abundant exosomes in the cervicovaginal lavage of women with cervical cancer. Abnormally high levels of microRNA-21 and microRNA-146a existed in the cervical cancer-derived exosomes and were functional in 293T cells.

## Supplementary Information



## Figures and Tables

**Table 1.** Cervical cancer-associated microRNAs used in this study.

MicroRNA	Validated methods	Validated targets	References
MicroRNA-21	Luciferase reporter assay	PDCD4	[10]
	RT-qPCR	PDCD4	[40]
	Northern blot analysis	/	[41]
MicroRNA-146a	Microarray/Northern blot	/	[42]
	RT-qPCR	ZNF813	[43]
	Microarray/qPCR	TRAF6/IRAK1	[44]
