# Supplementary material for: Increased Exosomal MicroRNA-21 and MicroRNA-146a Levels in the Cervicovaginal Lavage Specimens of Patients with Cervical Cancer
Source: Int J Mol Sci. 2014 Jan 8;15(1):758–73. doi: 10.3390/ijms15010758 (PMC3907836; doi:10.3390/ijms15010758)
Supplement: Supplementary file 1 [file ijms-15-00758-s001.pdf]

Supplementary Information

Figure S1. Enriched exosomal miR-146a and miR-21 in cervicovaginal lavage specimens of cervical cancer patients. We detrmined the miR-146a and miR-21 level in cervicovaginal lavage specimens of the normal subjects with HPV-negative (*n* = 32), subjects with HPV-positive (*n* = 25), or cervical cancer patients (*n* = 45). The relative amounts of miR-146a (**A**) or miR-21 (**B**) in the supernatant of specimens were examined by RT-qPCR; (**C**) and (**D**): The difference of miR-146a (**C**) or miR-21 (**D**) in the supernatant and in exosomes of specimens. The ratio of miR-146a or miR-21 in the supernatant to in exosomes in the normal HPV-negative group was designated as "1". All other samples were expressed as a relative value according to the ratio of in the supernatant to in exosomes. All experiments were performed in triplicate. Statistical significance was considered with a $p < 0.01$(**).

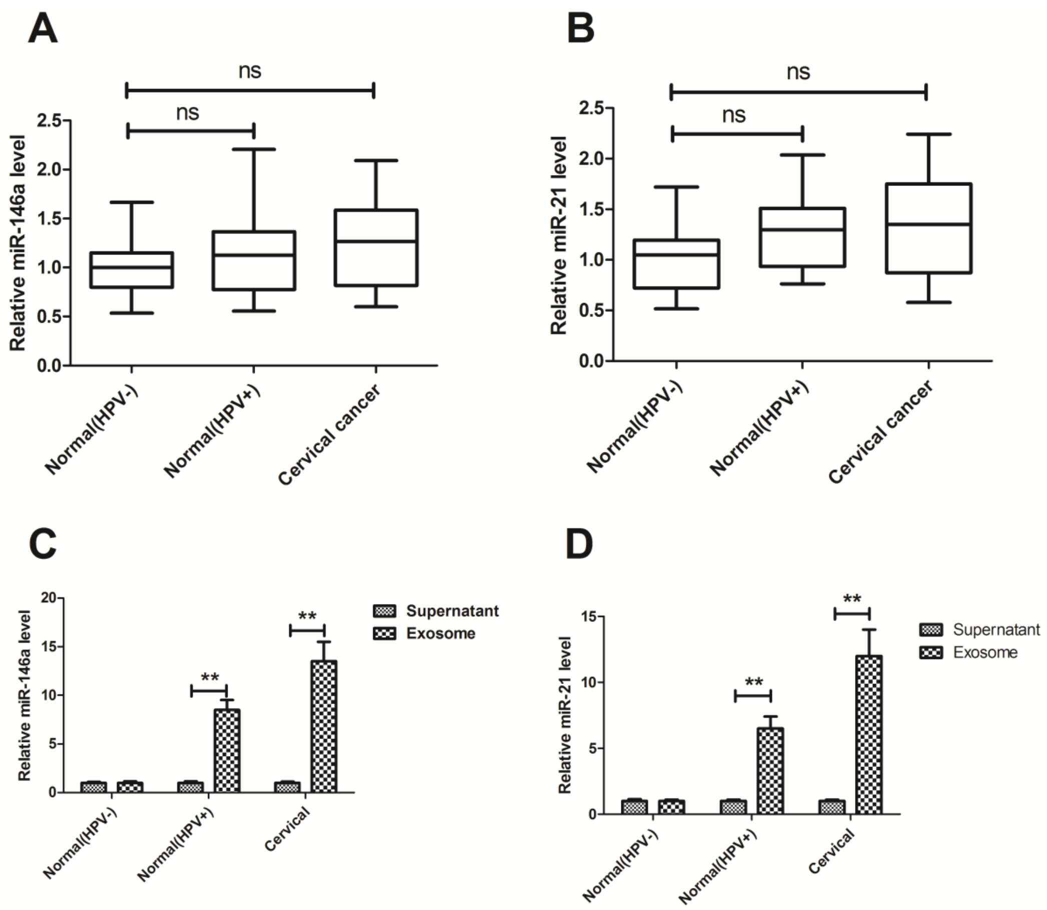

© 2014 by the authors; licensee MDPI, Basel, Switzerland. This article is an open access article distributed under the terms and conditions of the Creative Commons Attribution license (http://creativecommons.org/licenses/by/3.0/).
